# Commentary: New Variant of MELAS Syndrome With Executive Dysfunction, Heteroplasmic Point Mutation in the *MT-ND4* Gene (m.12015T>C; p.Leu419Pro) and Comorbid Polyglandular Autoimmune Syndrome Type 2

**DOI:** 10.3389/fimmu.2019.01333

**Published:** 2019-06-21

**Authors:** Josef Finsterer

**Affiliations:** Krankenanstalt Rudolfstiftung, Vienna, Austria

**Keywords:** heteroplasmy, mtDNA, oxidative phosphorylation, MELAS, stroke-like episode

With interest we read the article by Endres et al. about a 25 yo female with phenotypic features of a mitochondrial disorder (MID) in whom the mtDNA variant m.12015T>C in the *ND4* gene, occurring with a heteroplasmy rate of 12% (lymphocytes) respectively 15% (muscle) was made responsible for the phenotype (1). Clinical manifestations included stroke-like episodes (SLEs), dysexecutive syndrome (affective instability, sensory overload, disturbed concentration, impulse control problem), headache, myopathy, Hashimoto thyroiditis, hypocorticism, and other endocrine abnormalities (1). We have the following comments and concerns.

Assessment of the *ND4* variant m.12015T>C as pathogenic should be discussed. MtDNA variants are usually classified as pathogenic if they meet the criteria of the modified Yarham score (2). Items of the score are number of independent publications of the variant, heteroplasmy, segregation of the variant with the phenotype within a family, biochemical defect of complexes I, III, or IV, segregation of the variant with the biochemical defect in single fiber studies, evidence of pathogenicity of the variant in cybrid studies, evolutionary conservation of the nucleotide, and abnormal histopathology (2). If the score reaches 7–10 points, the variant is assessed as possible pathogenic, if reaching 11–13 points (without cybrid studies), the variant is assessed as probably pathogenic, and if reaching >11 points (with cybrid studies), the variant is definitively pathogenic (2). The variant m.12015T>C in the index article reaches 4 points and thus rather suggests a benign than a pathogenic variant.

A second point challenging pathogenicity of the variant is the low heteroplasmy rate in lymphocytes as well as muscle tissue (1). Phenotypic manifestation of a pathogenic mtDNA variant manifest usually not before the heteroplasmy rates exceed 50% in clinically affected tissues (3). Since the heteroplasmy rate was only 15% in muscle and the muscle was clinically affected, it is rather unlikely that the mtDNA variant was causative.

The diagnosis of a polyglandular inflammatory syndrome (1) is questionable, The patient had a history of Hashimoto thyroiditis but the autoimmune nature of Addison's disease is debatable. Since hypocorticism is a frequent manifestation of MIDs, particularly in patients with Kearns-Sayre syndrome (KSS) (4) and other MIDs (5, 6) and since no antibodies directed against adrenal components were found, the diagnosis a polyglandular autoimmune syndrome requires further discussion.

To confirm the presence of a stroke-like lesion (SLL) in the presented patient it would be helpful to see the ADC maps and perfusion studies of the cerebral lesion presented in Figure 2 (1). In the acute stage of a SLL DWI sequence and ADC map are usually hyperintens and perfusion studies generally show hyperperfusion (7). Additionally, the oxygen extraction is reduced within SLLs during the acute stage.

Overall, this interesting case report could be more meaningful if the pathogenesis of the mtDNA variant was assessed by application of the modified Yarham score, if the consequences of the low heteroplasmy rates were discussed, if MRI ADC maps and perfusion studies were provided, and if the classification of the patient as polyglandular autoimmune syndrome was revised. As long as the pathogenicity of the variant is not sufficiently proven, the conclusions drawn remain unsupported.

## Author Contributions

JF: design, literature search, discussion, first draft, critical comments.

### Conflict of Interest Statement

The author declares that the research was conducted in the absence of any commercial or financial relationships that could be construed as a potential conflict of interest.
